# Influence of Interactions among Polymeric Components of Automobile Shredder Residue on the Pyrolysis Temperature and Characterization of Pyrolytic Products

**DOI:** 10.3390/polym12081682

**Published:** 2020-07-28

**Authors:** Bin Yang, Ming Chen

**Affiliations:** School of Mechanical Engineering, Shanghai Jiao Tong University, No. 800, Dongchuan Rd., Shanghai 200240, China; robinshjt@sjtu.edu.cn

**Keywords:** automobile shredder residues (ASR), pyrolysis, co-pyrolysis, thermogravimetric analysis, gas chromatograph, polymer

## Abstract

Pyrolysis and gasification have gradually become the main means to dispose of automobile shredder residue (ASR), since these methods can reduce the volume and quality of landfill with lower cost and energy recovery can be conducted simultaneously. As the ASR pyrolysis process is integrated, the results of pyrolysis reactions of organic components and the interaction among polymeric components can be clarified by co-pyrolysis thermogravimetric experiments. The results show that the decomposition mechanisms of textiles and foam are markedly changed by plastic in the co-pyrolysis process, but the effect is not large for rubber and leather. This effect is mainly reflected in the pyrolysis temperature and pyrolysis rate. The pyrolytic trend and conversion curve shape of the studied ASR can be predicted by the main polymeric components with a parallel superposition model. The pyrolytic product yields and characterizations of gaseous products were analyzed in laboratory-scale non-isothermal pyrolysis experiments at finished temperatures of 500 °C, 600 °C, and 700 °C. The results prove that the yields of pyrolytic gas products are determined by the thermal decomposition of organic substances in the ASR and final temperature.

## 1. Introduction

With the rapid development of the economy in China, the quantity of cars has exceeded 260 million; thus, the number of end-of-life vehicles (ELVs) is also growing rapidly. Automobile shredder residue (ASR) accounts for approximately 20–25% of a vehicle by weight [1,2,3,4,5,6], which is considered an increasingly problematic mixture. Most industrialized countries require the recoverable and usable rate of automobiles to reach 95% [7,8,9,10]. The recycling of ASR has become indispensable for realizing these ELV recycling targets. Under environmental protection requirements and legal pressure, lots of research works and industrial applications have been conducted on the “3Rs” (reducing, reusing, and recovery) of ASR. Technologies for the treatment and disposal of ASR can be divided into the following kinds: (1) direct landfill; (2) material recovery, i.e., separation and collection technologies for material recovery based on the characteristics of color, density, magnetism, etc.; (3) and the thermal conversion of ASR [11,12]. Due to the growing shortage of land resources and potential soil pollution, increasing numbers of countries and regions are attempting to reduce ASR being used in landfill [8]. Commonly, ASR consists of plastics, textiles, leather, rubber, foam, wood, paper, glass, sand, metals, and other materials. Thus, the material recycling of ASR is complicated and costly due to the heterogeneous nature of the materials, the density, and the moisture content, which varies from site to site and from day to day as different types of automobiles are shredded [13]. Plastics, textiles, leathers, rubber, and foam are polymers that account for ~60–85% of the total weight of ASR [14,15]. Thermal conversion technologies can reduce the mass and volume of ASR, leading to the recovery of syngas, chemical building blocks, or fuel for energy production. Incineration, pyrolysis, and gasification are the most applied thermal technologies for ASR. Incineration or co-combustion in cement kilns can result in the emission of acidic gases, fine dust containing heavy metals, and organic pollutants, including polychlorinated dibenzo-ρ-dioxins and polychlorinated dibenzofurans [16]. Thus, pretreatment and pollution control are responsible for a huge proportion of the cost in the incineration of ASR. Pyrolysis and gasification have gradually become the main means to dispose of ASR as these methods can reduce the volume and quality of landfill with lower cost, while energy recovery can also be carried out simultaneously [17,18,19].

ASR pyrolysis processes produce solid, liquid, and gaseous products. In particular, pyrolysis oil and gas can be used as renewable alternative energy resources. High calorific value syngas (H_2_, CO and CH_4_) and bio-oil produced by ASR pyrolysis can be used for energy recovery [11,20]. Additionally, metal components in pyrolytic solid products can be recycled, where carbides can be used as fuel and inorganic materials in the production of building materials [21]. The ratios and characteristics of ASR pyrolytic products depend not only on the feed (e.g., fractions of organic versus inorganic), but also on the temperature, residence time, and other process parameters [18]. Galvagno et al. [22] evaluated both the process performance and characteristics of products obtained from the treatment of ASR using a pilot-scale experimental pyrolysis plant operating with different loads and varying process temperatures. Harder and Forton [23] analyzed variations in the physical characteristics of ASR, providing a review of the current technical developments in pyrolysis processes and emphasizing that the energy content of ASR is crucial to the design of a thermal process. Zolezzi et al. [18] evaluated the performance and product yields of conversional pyrolysis and fast pyrolysis of ASR at different pyrolysis temperatures. The study proved that the gas yield in conventional pyrolysis is higher than in fast pyrolysis, and higher heating values (HHV) of gas increase with an increasing temperature. Michele Notarnicola et al. [24] investigated the pyrolysis of ASR in a bench-scale rotary kiln at 450 °C, 550 °C, and 650 °C, focusing on the effect of temperature on product distribution and characterization.

Process parameters and pyrolytic products have been the main focus of research on ASR pyrolysis, but the interactions between components of ASR during pyrolysis have rarely been studied. However, compositions of ASR are highly heterogeneous, e.g., ~20–55% plastic, ~5–36% textiles, ~2–20% rubber, ~3–11% foam, ~1–15% metals, and ~10–20% fine materials (paint, glass, sand, etc.) [2,3,4,5,6,13,14,15]. During the pyrolysis process, each component decomposes at a different rate and temperature range due to its physicochemical properties. Thus, the variation in the main components of each different ASR source plays an important role in the ASR decomposition properties, and the total pyrolysis process of ASR is the integrated results of the pyrolysis reactions of the components involved in the process. Some research in the area of mixture co-pyrolysis and/or gasification has been conducted. By mixing plastics (without PVC) with biomass, the polymers increased activation energy bandwidth of biomass decomposition, the mass and heat transfer can be influenced, and thus the carbon conversion efficiency and volatiles yield will be increased [25,26,27,28,29,30,31]. During the co-pyrolysis of plastics and wood, properties of syngas generated from a mixed sample are not a weighted average of syngas properties obtained from separate gasification of each sample, which suggests synergistic effect due to co-gasification of wood and plastic [32,33]. PVC had strong interactions with rice, poplar wood, tissue paper, wool, terylene, and rubber powder during co-pyrolysis, the co-pyrolysis was usually promoted at low temperature, and the pyrolytic residue increased [34]. Haydary et al. [35] focused on the influence of the initial ASR composition on product yields, finding that the amount of char increased with increasing rubber content in the ASR and that gas products increased with increasing plastic materials content.

Clarifying the interaction between ASR components in the pyrolysis process would be beneficial for understanding the pyrolysis mechanisms of ASR and optimizing pyrolysis process parameters and product distribution. Focusing on this point, we conducted a series of pyrolysis experiments for ASR and its polymer components in this study. Aiming to analyze the co-pyrolysis behaviors of polymeric components, thermogravimetric (TG) analysis experiments for ASR were conducted, examining the main components and mixtures of different components. Product yields and characterizations of the pyrolytic gas products were investigated by non-isothermal pyrolysis experiments with an experimental laboratory-scale pyrolysis platform.

## 2. Experimental Procedure and Specimens

### 2.1. Experimental Specimens

The ASR specimens studied in this work were obtained from the Zhangjiagang Huaren Resources Recycling Co., Ltd. (Zhangjiagang, China), a domestic automobile dismantling enterprise. Reusable components with market value and particularly valuable material fractions such as batteries, air bags, tires, and catalytic converters are usually removed from ELVs. The remaining parts and the hulks are reduced to small pieces and most of the metal fraction is sorted using magnetic separation and eddy current separation, and the remaining fraction is called ASR, which amounts to ~20–25% of the average input ELV weight. ASR initial specimens were continuously obtained over one week from a crushing and sorting production line for ELVs. Visible bulks of metal to the human eye were sought out from the ASR initial specimens. The characteristics of the ASR specimens were determined by manual sorting and balancing by a Mettler Toledo analytical balance (Changzhou, China), and the results are shown in Table 1.

Let P, T, L, R, and F represent plastics, textiles, leather, rubber, and foam, respectively. P50R50 refers to a mixture composed of plastic and rubber, and their mass percentages are both 50%. Similar to this, P50L50 refers to a mixture composed of plastic and leather, P50T50 refers to a mixture composed of plastic and textiles, and P50F50 refers to a mixture composed of plastic and foam. MixASR refers to a mixture composed of plastic, rubber, leather, textiles and foam. The constituents of P50R50, P50L50, P50T50, P50F50, and MixASR are shown in Table 2. The mixtures were mixed well to obtain as homogeneous a mixture as possible. All specimens were combined and mixed well to ensure a representative sample for the determination, and uniformity of all specimens regarding the particle size (approximately ≤0.5 mm) was achieved. The reaction mechanism of ASR was unknown, so keeping the initial mass of each sample at a fixed constant for all measurements was safe [36]. Thus, the masses of all samples for the TG experiments were 10 ± 0.2 mg, considering the size of the sample pan and the accuracy of balance in the thermogravimetric analyzer, whereas the masses of all samples for the laboratory-scale pyrolysis experiments were recorded in 50 ± 0.2 g.

### 2.2. Experimental Procedure

Thermogravimetric (TG) analysis is one of the techniques most commonly used to explore the primary reactions of the decomposition of solids [37,38,39,40]. TG analysis measures the decrease in substrate mass caused by the release of volatiles during thermal decomposition as a function of time. TG analysis provides a rapid method for determining the thermal decomposition profile and significant parameters of the pyrolysis process, such as the temperature and retention time, which can be ascertained with TG experiments and by analyzing the pyrolysis kinetics [41,42,43,44]. Therefore, the pyrolysis kinetics of ASR have been widely studied using TG analysis methods [45,46]. The TG experiments were carried out with a thermogravimetric analyzer (Perkin-Elmer TGA 8000, Waltham, Massachusetts, United States) and the Pyris AutoStepwise TG software package (Perkin-Elmer, Waltham, Massachusetts, United States). The reaction evolution of plastics, textiles, rubber, leather, foam, P50R50, P50L50, P50T50, P50F50, ASR, and MixASR were recorded for a full range of temperatures from 30 °C to 800 °C at a constant heating rate of 10 °C/min under atmospheric control with high-purity (99.99%) nitrogen at a rate of 20.0 mL/min. The pressures of pneumatic gas and balance gas were set to 0.1 and 0.2 MPa, respectively. This nitrogen flow rate ensures an inert atmosphere for the sample during the run, whereas a small sample and slow heating rate ensures that the heat transfer limitations can be ignored.

**Figure 1:**
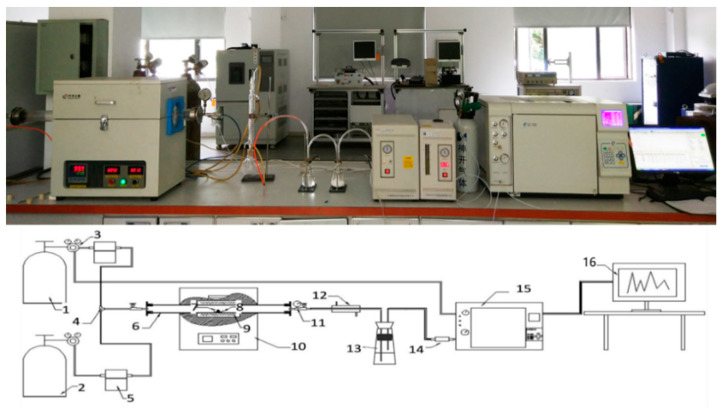
Experimental platform for laboratory-scale pyrolysis. 1: N2; 2: Other gases (air); 3: Pressure valve; 4: Three-way valve; 5: Gas flow controller; 6: Quartz tube; 7: Samples; 8: Crucible; 9: Heating unit; 10: Tubular pyrolysis furnace; 11: Pressure valve; 12: Condensing unit; 13: Acid-base gas cleaning unit; 14: Sampling valve; 15: The gas chromatograph; 16: Computer.

A laboratory-scale pyrolysis experimental platform was established, as shown in Figure 1. Pyrolytic product yields of plastics, textiles, rubber, leather, foam, P50R50, P50L50, P50T50, P50F50, ASR, and MixASR were calculated by non-isothermal pyrolysis experiments under atmospheric control with high-purity (99.99%) nitrogen at a rate of 20.0 mL/min. The heating rate of all experiments was 10 °C/min, and the finish temperatures were 500 °C, 600 °C, or 700 °C. The masses of solid products were weighed by a Mettler Toledo analytical balance (Changzhou, China) and the characterization of gas products were characterized with a GC-7860 gas chromatograph (Shanghai, China).

### 2.3. Kinetics Analysis of Co-Pyrolysis

According to Arrhenius law, the conversion form of the pyrolysis reaction rate of solid materials, when heating at a constant heating rate, can be expressed as follows [47]:

(1) $$\frac{d\alpha }{dT}=\frac{A}{\beta }\exp\left(-\frac{E}{RT}\right)f\left(\alpha \right)$$

where *A* and *E* are kinetic triplets, the pre-exponential factor ($s^{-1}$) and the activation energy ($J\cdot mol^{-1}$), respectively; β=dT/dt is the constant heating rate; R is the universal gas constant, 8.3145 $J\cdot mol^{-1}\cdot K^{-1}$; T is the absolute temperature, and K; *f*(*α*) denotes the reaction model which that depends on the conversion *α*, also known as the normalized mass (α∈[0,1]) of the released volatiles, which can be expressed as follows:

(2) $$\alpha =\frac{m_{0}-m_{t}}{m_{0}-m_{f}}$$

where *m*_0_, *m_t_*, and *m*_ꝏ_ denote the normalized mass of the sample at the initial temperature *t*_0_, given temperature *t*, and final temperature *t*_ꝏ_, respectively.

Considering the compositions of pyrolysis samples,

(3) $$m_{0}=(W_{1}+W_{2}+W_{3}+\cdots +W_{n})m_{0}$$

(4) $$m_{t}=(W_{1}wt_{1}+W_{2}wt_{2}+W_{3}wt_{3}+\cdots +W_{n}wt_{n})m_{0}$$

(5) $$m_{f}=(W_{1}wf_{1}+W_{2}wf_{2}+W_{3}wf_{3}+\cdots +W_{n}wf_{n})m_{0}$$

Then, Equation (2) can be arranged as:

(6) $$\begin{array}{ll} \alpha & =\frac{W_{1}(1-wt_{1})+W_{2}(1-wt_{2})+W_{3}(1-wt_{3})+\cdots +W_{n}(1-wt_{n})}{W_{1}(1-wf_{1})+W_{2}(1-wf_{2})+W_{3}(1-wf_{3})+\cdots +W_{n}(1-wt_{n})}\\ & =\frac{W_{1}\alpha_{1}(1-wf_{1})+W_{2}\alpha_{2}(1-wf_{2})+W_{3}\alpha_{3}(1-wf_{3})+\cdots +W_{n}\alpha_{n}(1-wf_{n})}{W_{1}(1-wf_{1})+W_{2}(1-wf_{2})+W_{3}(1-wf_{3})+\cdots +W_{n}(1-wt_{n})} \end{array}$$

For a kind of ASR, $W_{1},\;W_{2},\;W_{3},\ldots ,\;W_{n}$ are the initial mass percentages of each component in the ASR, which are constant and measured by manual sorting and balancing. For this, $wt_{1},wt_{2},wt_{3},\ldots ,wt_{n}$ are the dynamic weight rates during the pyrolysis process, whereas $wf_{1},wf_{2},wf_{3},\ldots ,wf_{n}$ are the final weight percentages measured by TG experiments for each component’s specimen, which are also constant. Since the composition of ASR is highly heterogeneous and the weight ratio of each component is uncertain for different ASR specimens, kinetics analysis for ASR is difficult. The conversion α of ASR is linearly dependent on conversions of each composition of ASR, such as plastic, textiles, leather, rubber, and foam. According to the composition analysis of ASR in Table 1, plastics, textiles, leather, rubber, and foam are the main components of ASR, accounting for 75.4% of the total weight. These polymers are the main reactants during ASR pyrolysis, so the Equation (6) can be simplified as:

(7) $$\alpha *=\frac{W_{P}\alpha_{P}(1-wf_{P})+W_{T}\alpha_{T}(1-wf_{T})+W_{L}\alpha_{L}(1-wf_{L})+W_{R}\alpha_{R}(1-wf_{R})+W_{F}\alpha_{F}(1-wf_{F})}{W_{P}(1-wf_{P})+W_{T}(1-wf_{T})+W_{L}(1-wf_{L})+W_{R}(1-wf_{R})+W_{F}(1-wf_{F})}$$

where $W_{P},\;W_{T},\;W_{L},\;W_{R},\;W_{F}$ denote the initial mass percentage of plastics, textiles, leather, rubber, and foam in an ASR specimen, respectively. Here, $wf_{P},\;wf_{T},\;wf_{L},\;wf_{R},\;wf_{F}$ are the final weight percentages measured by TG experiments for each specimen of plastics, textiles, leather, rubber, and foam, respectively. Here, ∆*α* is the difference value between *α* and α*. Considering the reaction model *f*(*α*) and kinetic triplets, such as the pre-exponential factor (A) and the activation energy (E), these can be calculated using conversion data. Therefore, the interaction mechanism between components during ASR pyrolysis may be demonstrated by comparing the linear superposition relationship of conversation with the experimental results based on TG experimental data.

## 3. Analysis of the Influence on Pyrolysis Process Parameters

### 3.1. Co-Pyrolysis of the Main Polymeric Components Based on TG Experiments

**Figure 2:**
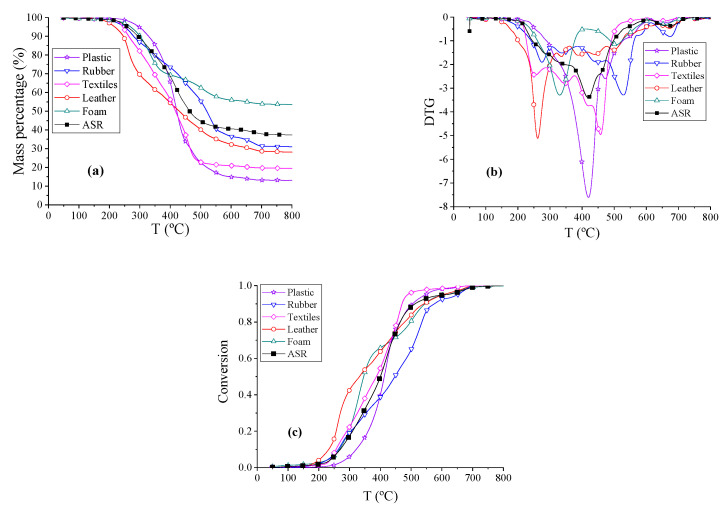
The thermogravimetric (TG), derivative thermogravimetry analysis (DTG), and conversion thermograms of automobile shredder residue (ASR) and its main components. (**a**) TG thermograms reflect the change in mass percentage during pyrolysis process. (**b**) DTG thermograms refers to the derivative curves of TG thermograms. (**c**) Pyrolysis conversion thermograms.

The thermogravimetry (TG) analysis curves, derivative thermogravimetry analysis (DTG) curves of ASR, and the pyrolysis of the five main components at a constant heating rate of 10 °C/min under a nitrogen atmosphere are shown in Figure 2a,b. Conversion (α) vs. temperature (T) “reaction profiles” for ASR and the main components of ASR were calculated with the TG data and the results are shown in Figure 2c. From these curves, we found that DTG curves of textiles, leather, rubber, and foam have two obvious peaks, whereas the DTG curves of plastic and ASR feature just one peak. There are more volatile products in the pyrolysis of plastics and textiles, but more solid products in the pyrolysis of foam and rubber. Let *T_d_*, *T_f_*, and *r*_ꝏ_ represent the starting temperature, the finishing temperature and the total weight loss rates, respectively. We defined the pyrolysis as the beginning when conversion α = 0.1 and the finish when α = 0.95, thus, non-isothermal pyrolysis yielding an 85% weight loss starts at temperature around *T_d_* and finishes at temperature around *T_f_*. The total weight loss rates *r*_ꝏ_, and the DTG peak temperatures, *T_d_* and *T_f_*, for the thermal decomposition processes are presented in Table 3. Combining Figure 2a–c, the weight percentages of samples fell quickly in the temperature range from 255 to 535 °C. The ranges of the pyrolysis temperature for actual ASR, MixASR, P50R50, P50L50, P50T50, and P50F50 were similar from 50 to 600 °C. Violent pyrolysis reactions of leather and textiles occurred at lower temperatures. The pyrolysis of ASR was not a simple superposition of the components, so it was necessary to analyze the interactions between the components in the pyrolysis process.

**Figure 3:**
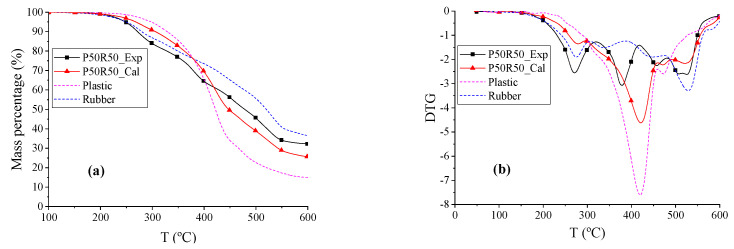
The TG and DTG thermograms of P, R, and P50R50. (**a**) TG thermograms reflects the change in mass percentage during pyrolysis process. (**b**) DTG thermograms refer to the derivative curves of TG thermograms.

The TG and DTG curves of plastic (P), rubber (R), and P50R50 are shown in Figure 3. The beginning reaction temperature *T_d_* of the blend P50R50 was 5.26 °C lower than R and 63.48 °C lower than P. The rubber radicals are hypothesized to catalyze the formation of radicals in plastic samples; this causes a positive synergistic effect in the system until the entire rubber sample is expected to pyrolyze completely [48]. The DTG curve of P50R50_Exp had three peaks, where the first peak and the third peak were mainly due to rubber pyrolysis, and the second peak was led by plastic pyrolysis. Within temperature ranges from 258.16 to 418.86 °C, the weight loss rate of P50R50_Exp was higher than P50R50_Cal, whereas the exact opposite was observed in temperature ranges from 418.86 to 600 °C. As can be seen from the temperature changes, rubber and plastics reduce each other’s pyrolysis temperature in co-pyrolysis process. However, the total weight loss rates *r*_ꝏ_ of P50R50_Exp were 6.55% higher than P50R50_Cal; thus, more solid phases were formed. The main reason for large amount of char is that rubber contains more inorganic impurities and black carbon, where a high ash content leads to more solid products [35]. This char of rubber pyrolysis hinders the pyrolysis of the plastic, and the volatiles formed are suspected to deposit in the char, which causes a negative synergy in the system [48,49]. The experimental curves do not fit well with the calculated curves; thus, the co-pyrolysis of plastic and rubber cannot be simply analyzed via a parallel superposition model.

**Figure 4:**
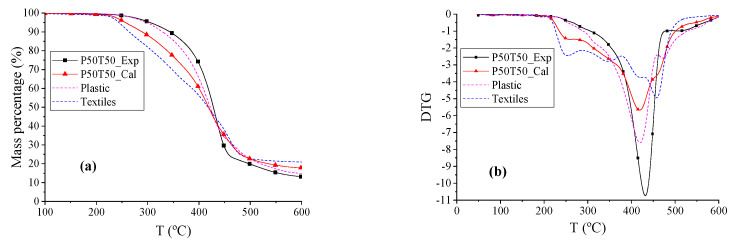
The TG and DTG thermograms of P, T, and P50T50. (**a**) TG thermograms reflects the change in mass percentage during pyrolysis process. (**b**) DTG thermograms refer to the derivative curves of TG thermograms.

The TG and DTG curves of plastics (P), textiles (T), and P50T50 are shown in Figure 4. The beginning reaction temperature Td of P50T50 was 78.74 °C higher than T, and 13.15 °C higher than P. The DTG peak temperatures and Tf of P, P50T50_Exp, and P50T50_Cal were similar, and the shape and overall trend of the P50T50_Exp and P50T50_Cal curves were also similar to that of P. Within temperature ranges from 248.95 to 385.37 °C, the weight loss rate of P50T50_Exp was lower than P50T50_Cal, whereas the exact opposite was observed in temperature ranges from 385.37 to 600 °C. When the temperature was below 385.37 °C, both textiles and plastics featured slow pyrolysis reactions. According to shape of the TG/DTG curves, the co-pyrolysis of plastic and textiles is dominated by plastic and produces more volatile products.

**Figure 5:**
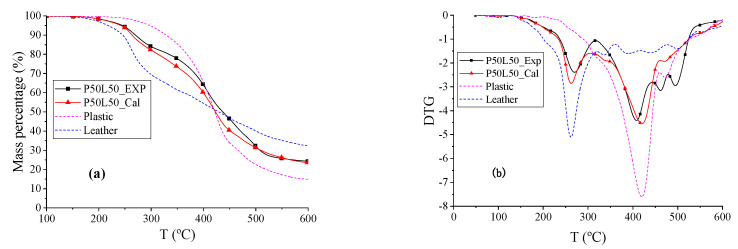
The TG and DTG thermograms of P, L, and P50L50. (**a**) TG thermograms reflects the change in mass percentage during pyrolysis process. (**b**) DTG thermograms refer to the derivative curves of TG thermograms.

The TG and DTG curves of plastic (P), leather (L), and P50L50 are shown in Figure 5. The beginning reaction temperature *T_d_* of the blend P50T50 was 27.47 °C higher than L and 61.88 °C lower than P. The experimental curves fit well with the calculated curves via the parallel reaction model. The DTG curve of blend P50L50 has two peaks. The temperature of the first peak is similar to the peak temperature of L, whereas the temperature of the second peak is similar to the peak temperature of P. The reaction rate of P50L50 was faster than plastic and lower than leather, and the total weight loss rates *r*_ꝏ_ of P50L50_Exp and P50L50_Cal were the same. Therefore, the co-pyrolysis of plastic and leather could be analyzed using the parallel superposition model.

**Figure 6:**
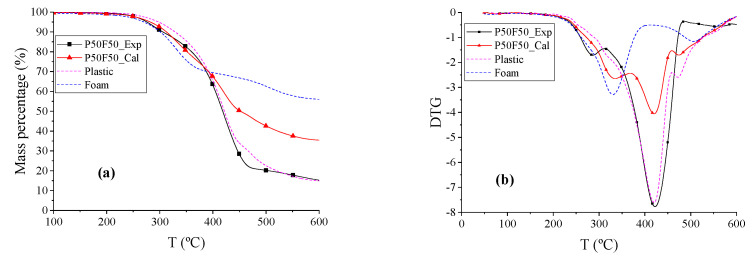
The TG and DTG thermograms of P, F, and P50F50. (**a**) TG thermograms reflects the change of mass percentage during pyrolysis process. (**b**) DTG thermograms refers to the derivative curves of TG thermograms.

The TG and DTG curves of plastic (P), foam (F), and P50F50 are shown in Figure 6. The beginning reaction temperature *T_d_* of the blend P50F50 was 28.84 °C higher than T and 25.63 °C lower than P. The DTG peak temperatures of P, P50F50_Exp, and P50F50_Cal are similar, and the shape and overall trend of the P50F50_Exp and P50F50_Cal curves are also similar to that of P, and the total weight loss rates of P50F50 and P were the same at 534.01 °C. Therefore, the co-pyrolysis of plastic and foam is dominated by plastic, and the decomposition of foam was increased and changed by plastic here.

**Figure 7:**
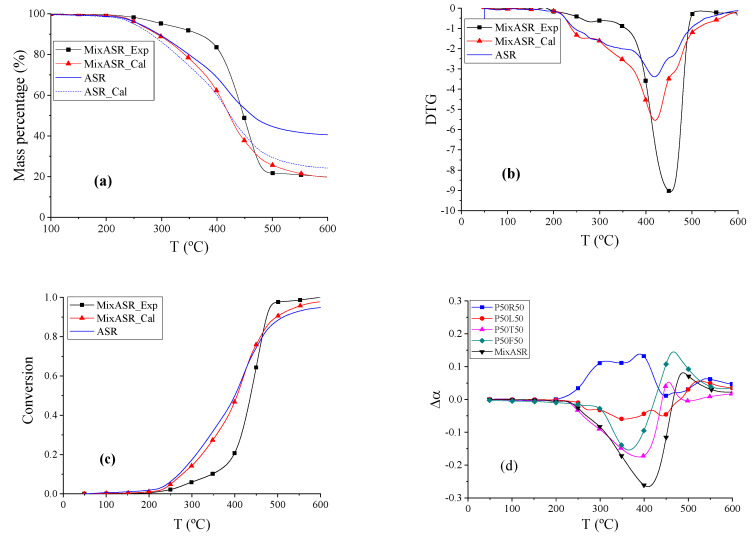
The TG, DTG, and conversion thermograms of MixASR and ASR and ∆α curves of the samples. (**a**) TG thermograms reflects the change in mass percentage during pyrolysis process. (**b**) DTG thermograms refer to the derivative curves of TG thermograms. (**c**) Pyrolysis conversion thermograms. (**d**) Conversion rate difference thermograms are subtracting the calculated value from the experimental value.

The TG, DTG, and conversion curves of actual ASR, MixASR_Exp, and MixASR_Cal are shown in Figure 7a–c. The beginning reaction temperatures *T_d_* of ASR, MixASR_Exp, and MixASR_Cal were 270 °C, 347.07 °C, and 277.69 °C, respectively. According to the data shown in Figure 7b, the DTG peak temperatures of ASR, MixASR_Exp, and MixASR_Cal were 418.76 °C, 453.97 °C and 420.22 °C, respectively. Within temperature ranges from 211.88 to 410.33 °C, the weight loss rate of MixASR_Exp was higher than MixASR_Cal, whereas the exact opposite was observed in temperature ranges from 410.33 to 600 °C, as shown in Figure 7a. The shape and overall trend of the conversion curves of Mix_ASR and ASR are similar. By fitting the TG data of ASR based on the material composition of ASR (shown as ASR_cal curve in Figure 7c), we found that the total weight loss rate *r*_ꝏ_ of the fitted TG curve was 75.7763% and the total weight loss rate *r*_ꝏ_ of MixASR was 80.1983%. The pyrolysis mechanism of the blend includes these five components and was inconsistent with parallel superposition model. However, the pyrolytic trend and conversion curve shape of MixASR_Cal and ASR were similar, so that the kinetic analysis of the studied ASR could be predicted with a parallel superposition model based on the TG experiments and analysis of each of the main components.

To compare the co-pyrolysis characteristics of various mixtures, including P50R50, P50T50, P50L50, P50F50, and MixASR, the data were further processed by subtracting the calculated value from the experimental value, and the results are shown in Figure 7d. The reaction trend of mixture P50R50 was different than MixASR, whereas the reaction trends of mixtures P50T50, P50L50, and P50F50 are similar to that of MixASR. Considering the compositions of the studied ASR (Table 1), rubber only accounts for 2.2%, and according to the above analysis, the co-pyrolysis characteristics of P50T50 and P50F50 were determined by plastic, where the co-pyrolysis of plastic and leather could be analyzed by the parallel superposition model, thus, linear superposition equations such as Equations (3)–(7) could be applied to predict the pyrolysis characteristics of ASR with the results of each of the components. According to the above analysis, the selection of the ASR pyrolysis temperature in the actual process can be determined based on the pyrolysis mechanism of plastic and textiles. This is mainly because they account for the largest proportion in the ASR and can influence the pyrolysis temperature of other polymeric components.

### 3.2. Pyrolytic Product Yields and Characterization of Gaseous Products

The pyrolytic product yields of plastic, textiles, rubber, leather, foam, P50R50, P50L50, P50T50, P50F50, ASR, and MixASR were obtained by the laboratory-scale pyrolysis experimental platform (Figure 1), and the results are shown in Table 4. With increasing finished pyrolysis temperature, the masses of solid and liquid products in all samples decreased, while gas products increased. Comparing the experimental results with the calculated results by the parallel superposition model of P50R50, P50L50, P50T50, P50F50, and MixASR, shown in Table 5, numerous trends can be observed. For the production of liquid products of P50R50, the experimental results were much lower than those calculated by the parallel superposition model. This was the same for the production of solid products from P50F50. This proved once again that interactions occur among the components in the pyrolysis process, rather than simple linear superposition reaction relationships. Particularly, the analysis of P50R50 and P50F50 using linear superposition theory had large errors. For pyrolytic product yields of MixASR at 500 °C and 600 °C pyrolysis temperatures, the differences between the experimental results and calculated results by the parallel superposition model were relatively small. These results were the same as the result of the TG analysis in the previous section. The yield of pyrolytic gas products was determined by the thermal decomposition of organic substances in the ASR and the final temperature. A similar trend was also observed in [35], where the polymeric fraction of ASR was pyrolyzed in a laboratory-scale screw type reactor under various conditions.

The characterizations of the gas products of P50R50, P50L50, P50T50, P50F50, ASR, and MixASR were analyzed by a gas chromatograph and the results are shown in Table 6. In terms of energy recovery and use, syngas with a high calorific value was expected to be a product of ASR pyrolysis. The experimental data showed that production of H_2_, CO, and CH_4_ was greatest at 600 °C. The characterizations of gas products of ASR and MixASR pyrolysis were consistent. However, the production of CO_2_ was always high, which would lead to a decrease in the calorific value of the syngas. Therefore, CO2 should be further eliminated and the calorific value of syngas should be improved. The total proportion of H2, CO, and CH4 in gas products of ASR pyrolysis exceeded 30% at finished temperatures of 600 °C and 700 °C. Therefore, pyrolysis synthesis of syngas is a promising direction for ASR recovery and treatment.

## 4. Conclusions

Our work consolidates the understanding of the synergistic effect between plastics, textiles, leather, rubber and foam, on pyrolysis temperature, product yields and gas species yields. Co-pyrolysis characteristics of five main components of ASR were investigated with TG experiments for ASR, each main component (plastics, textiles, leather, rubber, foam) and various mixed components via thermogravimetric experiments and laboratory-scale pyrolysis experiments under non-isothermal conditions. Analyzing the TG and DTG thermograms drawn by the Origin software package, the influence of the interactions among the polymeric components in terms of pyrolysis was discussed here, considering reaction temperature. The beginning reaction temperature of blends plastic-rubber, plastic-leather and plastic-foam were lower than that of plastic. The synergistic effect is reflected in the change of conversion rate and activation energy, and results showed that the decomposition mechanisms of textiles and foam were greatly changed by plastic during the co-pyrolysis process, while this was not significant for rubber and leather. The pyrolytic trend and conversion curve shapes of ASR and MixASR_Cal that were calculated by the parallel superposition model were consistent.

Pyrolytic product yields and characterizations of the gaseous products were analyzed by laboratory-scale pyrolysis experiments at finished temperatures of 500 °C, 600 °C, and 700 °C. With an increasing finished pyrolysis temperature, the yields of solid and liquid products of all samples decreased, while the masses of gas products increased. The total proportion of H_2_, CO, and CH_4_ in gas products of ASR pyrolysis exceeds 30% at finished temperatures of 600 °C and 700 °C, whereas the ratio of H2 and CO is close to one.

The synergistic effect observed by the thermogravimetric analysis agrees with that in laboratory-scale pyrolysis experiments. According to the analysis results, the pyrolysis temperature and pyrolytic product yields can be optimized by reducing non-polymer components and adjusting the ratio of different polymer components in the pretreatment process. Results showed liquid products are the most volatile products of ASR pyrolysis, most of which can be further converted into gas products. Aiming to obtain syngas with a greater calorific value, catalytic pyrolysis and gasification is an important research direction [50,51]. When ASR was pyrolyzed at finished temperatures of 600 °C under heating rate of 10 °C/min, the ratio of H_2_ and CO in the gas products is so good that the gas products can be processed into raw gas for hydrogen production.

## Figures and Tables

**Table 1:** Material compositions of the experimental specimens.

Sample	Material Composition						
	Metals	Plastics	Textiles	Leather	Rubber	Foam	Others
ASR	3.0	39.7	28.1	3.3	2.2	2.1	21.6
MixASR	0	53	37	3	4	3	0

**Table 2:** The constituents of mixed samples.

Samples.	P50R50	P50T50	P50L50	P50F50	MixASR
Components Ratio	P:R = 50:50	P:T = 50:50	P:L = 50:50	P:F = 50:50	P:T:R:L:F = 53:37:3:4:3

**Table 3:** The results of the weight loss rate and characteristics of the reaction temperature. Here, P, T, L, R, and F represent plastics, textiles, leather, rubber, and foam, respectively.

Materials	*r*_ꝏ_ (%)	Peak Temperature (°C)			*T_d_* (°C)	*T_f_* (°C)
P	86.7556	420.06	-		321.64	546
T	80.3423	348.39	457.46		256.05	489
L	71.4685	261.88	388.03		232.29	604.3
R	68.74	337.12	529.69		263.44	650.71
F	46.5	330.44	506.56		267.17	611.23
P50R50	67.8013	270.86	378.45	526.48	258.16	590.21
P50L50	75.7444	269.71	408.17		259.76	560.13
P50T50	86.8065	438.88	-		334.79	571.12
P50F50	84.7557	421.95	-		296.01	534.01
MixASR	80.1983	420.22	-		347.07	519
ASR	62.5385	418.76	-		270	569.4

**Table 4:** Distribution of pyrolytic products of different samples at different finished temperature.

Samples	500 °C			600 °C			700 °C		
	Solid	Liquid	Gas	Solid	Liquid	Gas	Solid	Liquid	Gas
P	22.23	65.01	12.76	14.88	65.29	21.83	13.21	64.35	22.44
R	55.59	35.98	8.43	36.52	34.73	28.75	31.35	31.82	36.83
T	22.57	65.79	11.64	20.91	60.34	18.75	19.72	60.09	20.19
L	40.12	50.15	9.73	32.17	56.24	11.59	28.64	57.23	14.13
F	62.59	24.91	12.50	55.97	17.31	26.72	53.95	17.33	28.75
P50R50	45.72	27.57	26.71	32.22	17.85	49.93	29.04	20.49	50.47
P50T50	19.71	70.56	9.73	13.19	65.34	21.47	12.13	57.94	29.93
P50L50	31.95	61.17	6.88	24.26	57.32	18.42	21.95	55.48	24.33
P50F50	20.19	52.73	27.08	15.27	45.94	38.79	14.56	42.82	42.62
MixASR	21.71	62.68	15.61	19.79	54.37	25.84	17.11	48.76	34.13
ASR	44.71	43.48	11.81	40.56	39.68	19.76	37.89	34.77	27.34

**Table 5:** The differences between the experimental results and the calculated results.

T (°C)	Products	P50R50	P50T50	P50L50	P50F50	MixASR
500 °C	Solid	6.81	−2.69	0.78	−22.22	−3.57
	Liquid	−22.93	5.16	3.59	7.77	0.05
	Gas	16.12	−2.47	−4.37	14.45	3.52
600 °C	Solid	6.52	−4.71	0.73	−20.16	0.11
	Liquid	−32.16	2.53	−3.45	4.64	−6.37
	Gas	24.64	1.18	1.71	14.52	5.20
700 °C	Solid	6.76	−4.34	1.03	−19.02	−0.89
	Liquid	−27.60	−4.28	−5.31	1.98	−11.34
	Gas	20.84	8.62	6.05	17.03	12.23

**Table 6:** The proportion of the main small molecule gases in the pyrolytic gas products.

Samples	T (°C)	H_2_	CO	CO_2_	CH_4_	C_2_H_4_	C_2_H_6_	C_3_H_6_	C_3_H_8_
P50R50	500	3.74	4.63	24.53	3.36	1.46	1.53	1.04	0.38
	600	7.05	8.13	19.14	7.34	2.18	2.58	1.86	0.67
	700	10.62	9.15	17.64	10.16	0.31	1.84	2.79	0.42
P50T50	500	5.84	7.19	30.18	7.53	0.67	1.28	1.01	0.54
	600	11.42	13.46	18.27	12.37	1.64	2.47	0.64	0.12
	700	13.17	15.74	24.16	6.18	2.08	3.14	0.58	0.34
P50L50	500	5.66	10.39	22.90	8.78	1.23	1.70	0.41	0.30
	600	10.11	17.85	30.63	11.34	0.59	1.10	0.56	0.25
	700	6.07	16.14	19.88	7.59	1.88	1.05	0.77	0.49
P50F50	500	6.74	6.62	16.34	9.70	0.34	1.48	2.34	0.35
	600	8.76	9.14	20.34	10.11	1.23	1.79	3.04	0.24
	700	10.92	11.47	11.54	4.87	7.94	1.04	2.17	0.37
MixASR	500	6.19	5.57	15.09	6.25	0.02	0.58	0.14	0.04
	600	10.11	13.85	20.63	11.34	0.59	1.10	0.56	0.25
	700	13.78	15.64	14.76	13.47	1.14	1.56	0.84	0.97
ASR	500	6.04	2.77	11.64	7.83	1.52	0.18	0.71	0.24
	600	11.47	9.68	20.70	10.18	0.22	0.29	0.39	0.23
	700	8.65	5.32	15.83	7.99	0.22	0.25	0.24	0.15

## Abbreviations

The following abbreviations are used in this manuscript:

ASR	Automobile shredder residues
MixASR	Mixtures of plastic, textiles, leather, rubber, and foam
TG	Thermogravimetric
DTG	Derivative thermogravimetry
P	Plastic
T	Textiles
L	Leather
R	Rubber
F	Foam
